# Relevance of pleural adhesions for short- and long-term outcomes after lung volume reduction surgery

**DOI:** 10.1016/j.xjon.2023.06.018

**Published:** 2023-07-14

**Authors:** Claudio Caviezel, Medea Rodriguez, Pavel Sirotkin, Ulrike Held, Isabelle Opitz, Didier Schneiter

**Affiliations:** a Department of Thoracic Surgery, University Hospital Zürich, Zürich, Switzerland; b Epidemiology, Biostatistics, and Prevention Institute, University of Zürich, Zürich, Switzerland

**Keywords:** LVRS, emphysema, pleura, adhesions

## Abstract

**Objective:**

Pleural adhesions (PLAs) have been shown to be a possible risk factor for air leak after lung volume reduction surgery (LVRS), but the relevance of PLA for lung function outcome remains unclear. We analyzed our LVRS cohort for the influence of PLA on short-term (ie, prolonged air leak) and long-term outcomes.

**Methods:**

Retrospective observational cohort study with 187 consecutive patients who underwent LVRS from January 2016 to December 2019. PLA were defined as relevant if they were distributed extensively at the dorsal pleura; were present in at least at 2 areas, including the dorsal pleura; or present extensively at the mediastinal pleura. In patients with bilateral emphysema, bilateral LVRS was performed preferentially. The objectives were to quantify the association of PLA and rate of prolonged air leak (chest tube >7 days), and the association of PLA with postoperative exacerbations and with forced expiratory volume in 1 second 3 months postoperatively. The associations were quantified with odds ratios for binary outcomes, and with between-group differences for continuous outcomes. To account for missing observations, 100-fold multiple imputation was used.

**Results:**

PLAs were found in 46 of 187 patients (24.6%). There was a 32.6% rate of prolonged air leak (n = 61), mean chest tube time was 7.84 days. A total of 94 (50.3%) LVRSs were unilateral and 93 were bilateral. There was evidence for an association between PLA and the rate of prolonged air leak (odds ratio, 2.83; 95% CI, 1.36 to 5.89; *P* = .006). There was no evidence for an association between PLA and postoperative exacerbations (odds ratio, 1.11; 95% CI, 0.5 to 2.45; *P* = .79). There was no evidence for an association between PLA and forced expiratory volume in 1 second (estimate −1.52; 95% CI –5.67 to 2.63; *P* = .47). Both unilateral and bilateral LVRS showed significant postoperative improvements in forced expiratory volume in 1 second by 27% (8.43 units; 95% CI, 3.66-13.12; *P* = .0006) and by 28% (7.87 units; 95% CI, 4.68-11.06; *P* < .0001) and a reduction in residual volume of 15% (−33.9 units; 95% CI, −56.37 to −11.42; *P* = .003) and 15% (−34.9 units; 95% CI, −52.57 to −17.22; *P* = .0001), respectively.

**Conclusions:**

Patients should be aware of potential prolongation of hospitalization due to PLA. However, there might be no relevant influence of PLA on lung function outcomes.

## Graphical abstract

**Figure:**
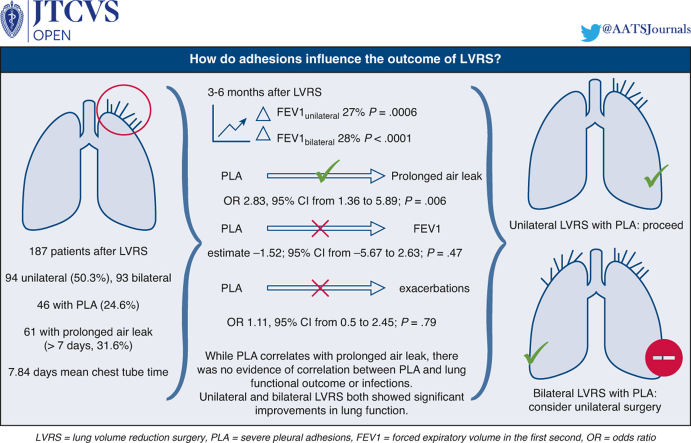

**Figure:**
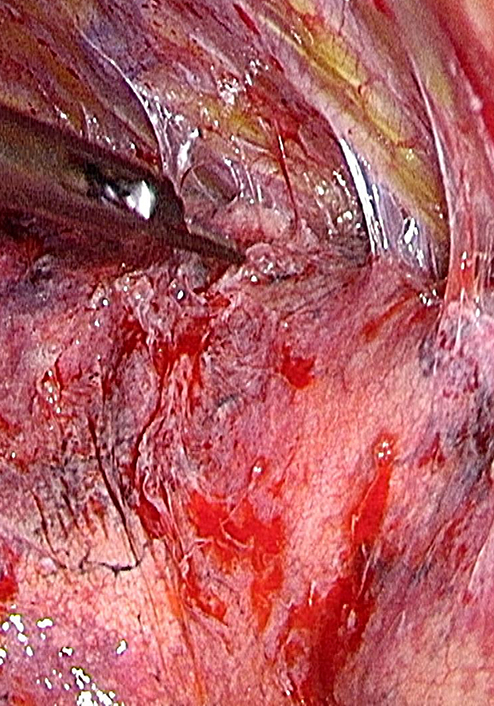
Thoracoscopic adhesiolysis of severe pleural adhesions.

**Central Message**

Pleural adhesions might prolong hospitalization after LVRS, but do not seem to influence pulmonary function outcomes.

**Perspective**

Patients with LVRS are fragile and it is important to know whether adhesiolysis of severe pleural adhesions, which leads to potential prolongation of hospitalization and subsequent risk of complications, has an influence on outcomes. This study confirms prolonged chest tube time after LVRS with severe adhesions, but finds no correlation between adhesiolysis and pulmonary function outcomes.

Lung volume reduction surgery (LVRS) has been shown to improve lung function, exercise capacity, and even survival in highly selected patients with severe emphysema.^1^^,^^2^ The only available randomized evidence deals with a lot of open surgery cases, in a study conducted during the late 1990s and early 2000s.^3^ With the advent of video-assisted thoracic surgery, the surgical approach has become much less harmful, thus reducing operative morbidity, especially by allowing earlier recovery at lower cost.^3^^,^^4^ Nevertheless, candidates eligible for LVRS still represent among the most vulnerable patient groups within the thoracic surgery community.^5^ Short operation times and short length of hospital stays are preferable to decrease the risk of postoperative complications such as delirium and hospital-acquired pneumonia.^6^ In most thoracic surgery procedures, and especially in LVRS, by far the most frequent postoperative complications are prolonged air leak (PAL) and the need for chest tube drainage.^7^ Data from the randomized National Emphysema Treatment Trial (NETT) were analyzed regarding postoperative air leaks and their associated risk factors. In these 552 patients, 90% developed an air leak, with a mean duration of 7 days. Not surprisingly, severe pleural adhesions (PLA) were a risk factor, as were low diffusion capacity (DLCO), low values of forced expiratory volume in 1 second (FEV1), and patients using inhaled steroids. Regarding PLA, there is no standardized definition to grade severity. However, PLA seem to prolong the operation itself, increase the risk of PAL postoperatively, and thus keep the patient in hospital for a longer time. Whether PLA also alter the outcome of LVRS in the long term has not yet been investigated. Long-term outcomes of LVRS might be measured by monitoring increase in lung function performance because this is the main goal of the procedure. Our group has long wondered whether longer hospital stays due to PAL, sometimes even accompanied by revision surgery, influence the targeted outcome of emphysema surgery 3 months postoperatively. If this was the case, a surgeon may decide to abort surgery after spotting severe PLA. Especially in bilateral LVRS, after 1 side has already undergone operated, PLA might be a reason to halt the procedure if explorative thoracoscopy uncovered PLA on the second side. We hypothesize poorer lung function outcome (LFO) 3 months postoperatively after LVRS accompanied by adhesiolysis of severe PLA.

## Methods

A retrospective observational cohort study was conducted, with 187 consecutive patients who underwent LVRS at our institution from January 2016 to December 2019. The time frame was chosen due to the availability of standardized information on PLA from 2016 onward and the initiation of a Masters thesis (by M.R.) during 2020.

### Patient Selection

Inclusion criteria for LVRS are listed in Table 1. All patients who were potential candidates for LVRS were discussed by our interdisciplinary emphysema board.

**Table 1:** Inclusion and exclusion criteria for lung volume reduction surgery

Criteria	Inclusion	Exclusion
Patient	Nicotine abstention >4 mo	Daily steroid intake >20 mg
CT morphology	Lung emphysema	Significant bronchiectasis
Lung function	FEV1 <45% TLC >100% RV >150%	FEV1 <20% and DLCO <20% in homogeneous emphysema
6-MWD	<450 m	–
Gas exchange		paco_2_ >6.7 Pa pao_2_ <6.0 Pa in homogeneous emphysema

*CT*, Computed tomography; *FEV1*, forced expiratory volume in 1 second; *TLC*, total lung capacity; *RV*, residual volume; *DLCO*, diffusion capacity; *6-MWD*, 6-minute walking distance.

### Operation

LVRS was preferentially performed bilaterally. The decision to perform a bilateral or unilateral operation depends primarily on the predominance of disease distribution. Additionally, besides their emphysema morphology, patients with borderline inclusion criteria, such as DLCO <20% or mild-to-moderate pulmonary hypertension, endobronchial valves in situ (on the nonoperated side), suspicious nodule/proven lung cancer planned for concomitant resection, and patients who had already had a thoracic operation (eg, LVRS, pleurodesis, or anatomical resection) on the other side, were operated unilaterally.

Target areas on computed tomography scans, combined with perfusion scintigraphy scans, were resected with standard staplers (Endo GIA Ultra Universal; Medtronic). In the case of macroscopically extremely fragile-looking tissue, we buttress the stapling lines. This practice is at the surgeon's discretion.

PLA were completely freed to mobilize the whole lung. In case of severe adhesions, a Ligasure Maryland tool (Medtronic) is sometimes used, as well as a monopolar cautery hook. However, if possible, we try not to use energy-requiring devices because the closer they come to lung tissue, the more the heat they generate, potentially creating predetermined breaking points. We therefore usually use scissors only as much as possible. In case of severe air leak at the end of the procedure, we might staple the lesion, and add Tachosil (Takeda) and/or Progel (Becton, Dickinson and Company). Other measures; for example, pleural tents or pleurodesis, are never used.

### Definition of PLA

**Figure 1:**
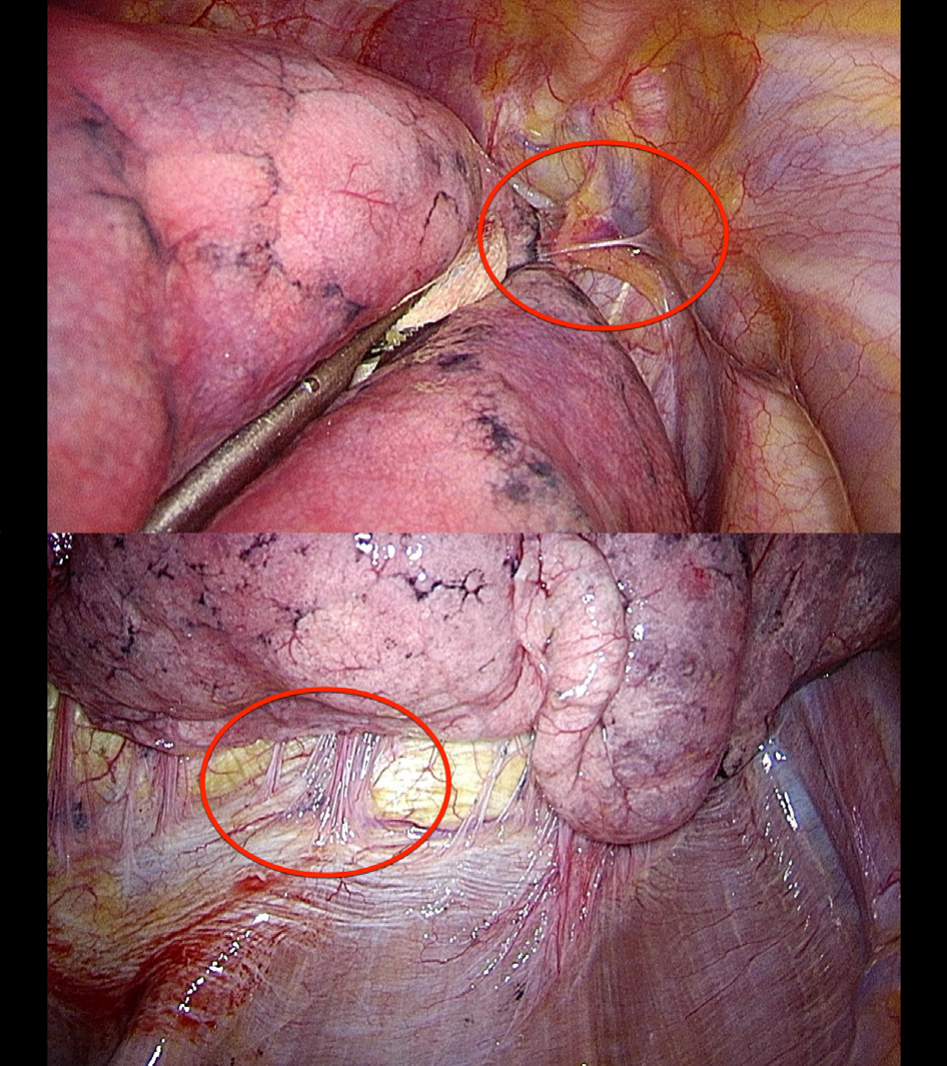
Nonsevere pleural adhesions. *Top*, *Red circle* indicates a singular adhesion between the apex of the right lung and the upper mediastinum. *Bottom*, Several nonsevere pleural adhesions between the right lower lobe and the diaphragm. Both pictures were taken during a lung volume reduction surgery procedure.

**Figure 2:**
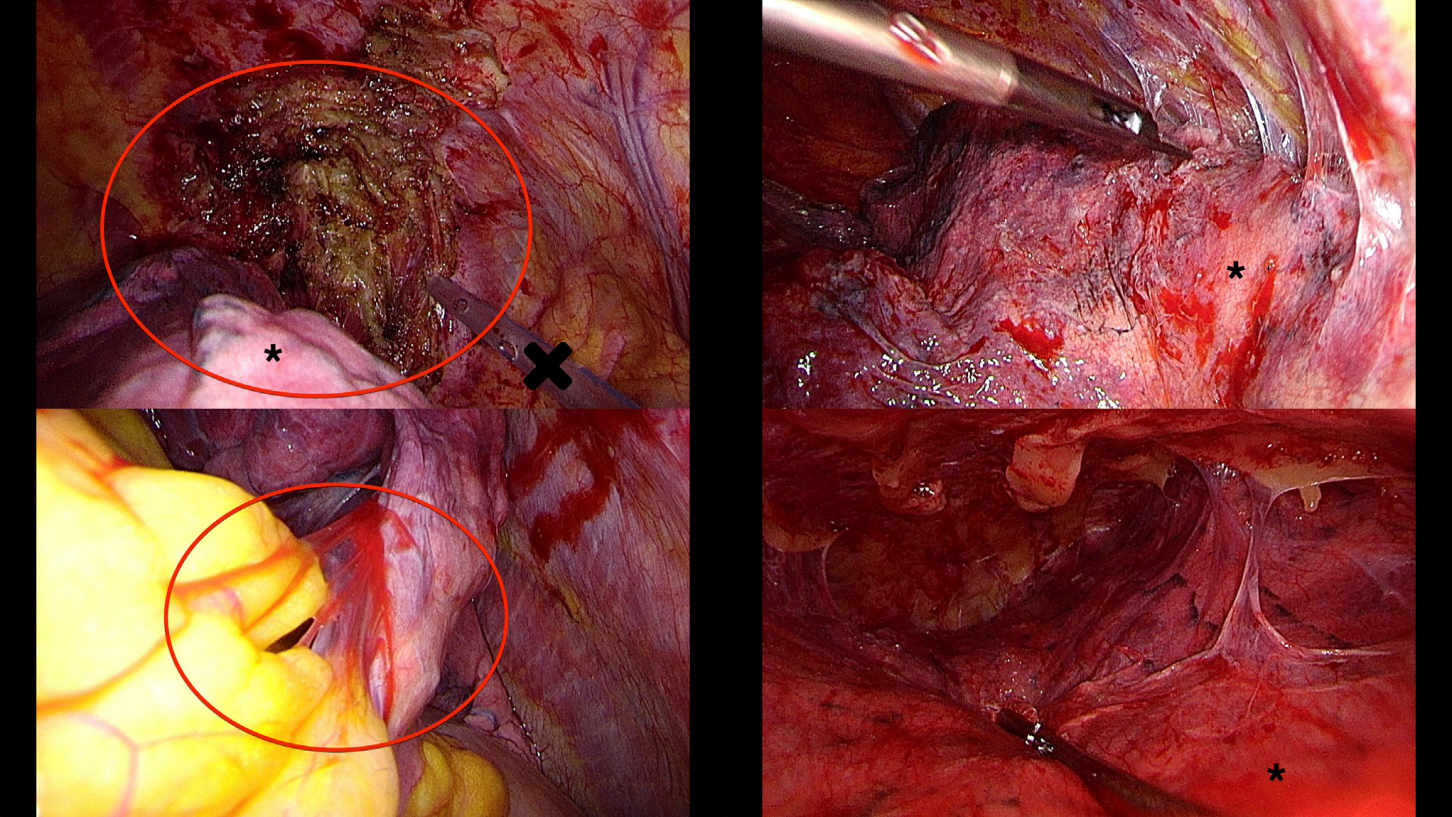
Severe pleural adhesions. *Top left*, Scar at the apical dorsal pleura (*red circle*), indicating expleural adhesiolysis—the adhesions included the basal upper lobe. *Top right*, Severe adhesions between sternum and right upper lobe. *Bottom left*, Adhesions between lingula and paracardial fatty tissue (*red circle*). *Bottom right*, Complete adhesive right pleural cavity; this operation was terminated without lung volume reduction surgery (LVRS) on this side after successful LVRS on the contralateral side. ∗Lung. †Chest tube.

In 2016, a standardized definition of PLA was introduced and applied prospectively by noting it in the operation report. Severe PLA were defined as relevant if they were distributed extensively at the dorsal pleura; in at least at 2 areas, including the dorsal pleura; or extensively at the mediastinal pleura (Figure 1 shows nonrelevant/nonsevere PLA, whereas Figure 2 shows relevant severe PLA). Please refer to Video 1 for an example of severe PLA in LVRS.

### Follow-up and Outcome Measures

All pulmonary function tests were performed using a standard body plethysmograph and DLCO. At our institution, most patients are monitored for only 3 months after LVRS. Patients from other institutions were sent back for follow-up and their referring physicians were asked to perform the pulmonary function tests. Because PAL (chest tube >7 days) and, in our setting, prolonged hospitalization (patients are not dismissed until tube-free) are the main and almost sole postoperative complications—even in borderline patients with mild-to-moderate pulmonary hypertension and with very low DLCO—these were regarded as short-term outcomes.^8^^,^^9^ Lung function performance is an important and, in retrospective analyses, easily measurable parameter and was therefore regarded as a long-term outcome.

More frequent chronic obstructive pulmonary disease (COPD) exacerbations (pulmonary exacerbations [PE]) were chosen as a documented parameter for another possible complication after hospitalization because there was no documented case of pneumonia or empyema in the patient group analyzed. Because 70% to 80% of COPD exacerbations are due to respiratory infections (one-third to two-thirds due to viruses, and one-third to one-half due to bacteria), some might be triggered by prolonged hospitalization.^10^

### Objectives

The primary hypothesis was poorer LFO 3 months postoperatively after LVRS accompanied by adhesiolysis of severe PLA. Therefore, we aimed to quantify the association of PLA with lung function (ie, FEV1%) 3 months postoperatively.

Besides collecting patient demographic characteristics and perioperative data, further objectives were to quantify:

• The association of PLA and rate of PAL (chest tube >7 days);
• The association of PAL with PE;
• The change in lung function 3 months postoperatively in uni- and bilateral surgery; and
• The association of PLA and length of stay in hospital (days).

### Statistical Methods

Associations of the binary independent variables of interest were quantified with odds ratios (OR) for binary outcomes, and with between-group differences for continuous outcomes. In a first step, unadjusted associations were estimated and reported. In a second step, adjusted associations were estimated and reported. All estimates were reported with 95% CI and exploratory *P* values. The confounding variables accounted for in this context were location, baseline FEV1, sex, and age. To account for missing observations, 100-fold multiple imputation was used. All analyses were performed with the programming language R (R Foundation for Statistical Computing), in combination with dynamic reporting using *knitr*.

### Ethics

This study was approved by the Swiss local ethics committee (No. KEK #2016 to 00716), last updated by the committee on November 24, 2020. Beginning in 2016, written consent is standardized for publication of patient data and is available for all patients.

## Results

**Figure 3:**
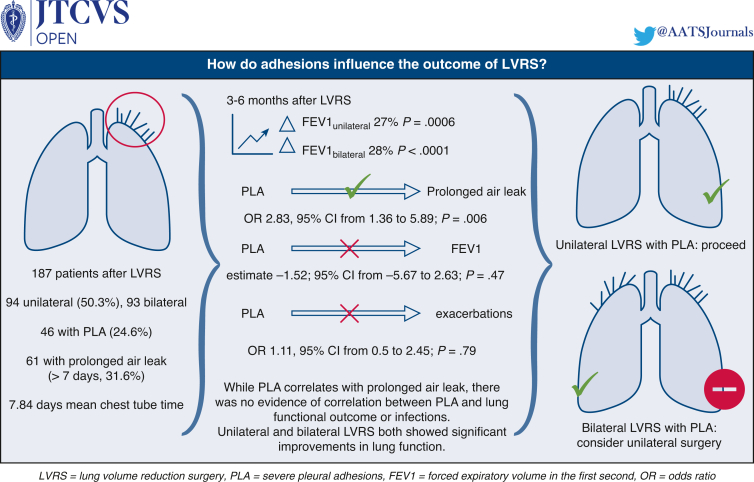
Summary of the study. *LVRS*, Lung volume reduction surgery; *FEV1*, forced expiratory volume in 1 second; *PLA*, severe pleural adhesions; *OR*, odds ratio; *CI*, confidence interval.

Within the study time period, 187 consecutive patients underwent LVRS at our institution. Table 2 shows baseline demographic characteristics of age, sex, lung function values, and whether bilateral or unilateral LVRS was performed. The graphical abstract presents a summary of the study (Figure 3).

**Table 2:** Patient demographic characteristics

Criteria	Overall	No adhesions	PLA	SMD	Missing (%)
Patients	187	141	46		
Age (y)	65.24 ± 8.45	65.23 ± 8.49	65.26 ± 8.43	0.004	0
Female	78 (41.7)	64 (45.4)	14 (30.4)	0.312	0
FEV1 predicted (%)	29.71 ± 10.28	29.06 ± 8.64	31.80 ± 14.25	0.232	1.1
TLC predicted (%)	129.06 ± 24.50	128.38 ± 25.19	131.30 ± 22.19	0.123	3.2
RV predicted (%)	226.61 ± 65.24	225.59 ± 67.09	229.99 ± 59.32	0.069	3.2
RV/TLC (%)	65.88 ± 10.47	66.00 ± 10.19	65.47 ± 11.50	0.048	3.7
DLCO predicted (%)	32.33 ± 11.53	32.01 ± 10.88	33.35 ± 13.47	0.109	4.3
Bilateral LVRS (%)	93 (49.7)	80 (56.7)	13 (28.3)	0.602	0
Unilateral LVRS (%)	94 (50.3)	61 (43.3)	33 (71.7)	0.602	0

Values are presented as mean ± SD or n (%) unless otherwise noted. *PLA*, Severe pleural adhesions; *SMD*, standardized mean difference; *FEV1*, forced expiratory volume in 1 second; *TLC*, total lung capacity; *RV*, residual volume; *DLCO*, diffusion capacity; *LVRS*, lung volume reduction surgery.

### Perioperative Outcomes

A subset of 14 patients underwent thoracotomy; all others (173 [92.5%]) were operated on using video-assisted thoracic surgery. A total of 94 patients (50.3%) were operated unilaterally: 37 (39.7%) due to disease distribution, 15 (15.9%) due to history of contralateral operation, 12 (12.8%) due to endobronchial valves in situ, 11 (11.7%) due to concomitant nodule/cancer resection, and 7 (7.4%) because of borderline inclusion criteria. Twelve patients (12.8%) were planned as bilateral LVRS, but the operation was terminated after the first side due to PLA with severe air leak.

Regarding morphology, 142 (75.9%) patients showed heterogeneous and 45 (24%) nonheterogeneous emphysema. Mean chest tube time for all patients was 7.8 ± 7.2 days. The rates of PLA and PE were 24.6% (46 patients) and 27.4% (51 patients), respectively.

### Association of PLA With PAL

There was strong evidence for an association between PLA and PAL; the unadjusted OR was 2.71 (95% CI, 1.36-5.41; *P* = .005). The adjusted OR of adhesions was 2.83 (95% CI, 1.36-5.89; *P* = .006).

### Association of PAL With PE

There was no evidence for an association between PAL with PE (OR, 1.65; 95% CI, 0.84-3.32; *P* = .15). This result remained unchanged after adjusting for confounders.

### Lung Function

Spirometric and bodyplethysmographic lung function values are displayed in Table 3. Both unilateral and bilateral LVRS showed significant postoperative improvements in FEV1 by 27% (+8.43 units; 95% CI, 3.66 to 13.12; *P* = .0006) and 28% (7.87 units; 95% CI, 4.68 to 11.06; *P* < .0001) and a reduction in residual volume by 15% (−33.9 units; 95% CI, −56.37 to −11.42; *P* = .003) and 15% (34.9 units; 95% CI, −52.57 to −17.22; *P* = .0001), respectively.

**Table 3:** Spirometric and body plethysmographic outcomes

Criteria	Overall	Bilateral	Unilateral	SMD	Missing (%)
n	187	93	94		
FEV1 before surgery predicted (%)	29.71 ± 10.28	27.92 ± 7.79	31.52 ± 12.07	0.354	1.1
FEV1 after 3 mo predicted (%)	37.89 ± 15.06	35.79 ± 11.81	39.95 ± 17.53	0.278	22.5
TLC before surgery predicted (%)	129.06 ± 24.50	130.85 ± 21.03	127.25 ± 27.57	0.147	3.2
TLC after 3 mo predicted (%)	123.04 ± 19.36	124.77 ± 19.61	121.27 ± 19.09	0.181	28.3
RV before surgery predicted (%)	226.61 ± 65.24	231.29 ± 53.44	221.89 ± 75.33	0.144	3.2
RV after 3 mo predicted (%)	192.25 ± 61.81	196.39 ± 57.46	187.99 ± 66.17	0.136	28.3
RV/TLC before surgery predicted (%)	65.88 ± 10.47	67.05 ± 7.35	64.70 ± 12.80	0.225	3.7
RV/TLC after 3 mo predicted (%)	60.03 ± 17.10	58.41 ± 9.37	61.69 ± 22.41	0.191	28.3
DLCO before surgery predicted (%)	32.33 ± 11.53	32.69 ± 9.90	31.97 ± 13.05	0.062	4.3
DLCO after 3 mo predicted (%)	37.21 ± 12.43	37.24 ± 11.43	37.18 ± 13.40	0.005	27.8

Values are presened as mean ± SD. *SMD*, Standardized mean difference; *FEV1*, forced expiratory volume in 1 second; *TLC*, total lung capacity; *RV*, residual volume; *DLCO*, diffusion capacity; *LVRS*, lung volume reduction surgery.

Results from the 6-minute walking distance (6-MWD) test were available preoperatively in 161 patients (86.1%) and postoperatively in 130 (69.5%). Preoperative mean distance was 300 ± 110 m, postoperative mean distance was 375 ± 106 m (*P* < .001).

### Association of PLA With Lung Function

There was no evidence for an association between PLA and FEV1 at 3-month follow-up when adjusting for baseline FEV1 (estimate, 2.34; 95% CI, −3.08 to 7.77; *P* = .39). The results remained unchanged when adjusting for confounders.

### Association of PLA and Length of Stay in Hospital

There was evidence for an association between length of stay in hospital (days) and the presence of adhesions (estimate, 3.83 days; 95% CI, 1.4-6.25; *P* = .002).

## Discussion

This retrospective observational cohort study found evidence of a correlation between PLA and PAL but no evidence of a correlation between PLA and functional outcome of LVRS; patients with PLA showed the same improvement in FEV1 3 months postoperation as patients without PLA. Neither was there evidence of correlation between PLA and postoperative COPD exacerbations.

Because this study again confirmed the known correlation between PLA and PAL with subsequent prolongation of hospitalization, our group feared that there might be a lesser functional benefit in the long term.^7^ Reasons for this included the possible increased risk of infectious complications and/or missed, or at least postponed, initiation of efficient mobilization and rehabilitation.

Regarding length of hospital stay versus PAL as outcome measurements, there are differing views. In our practice, patients have to stay in hospital for a direct transfer to inpatient postoperative rehabilitation. Nowadays, a lot of patients still go to rehabilitation, but a growing number are discharged directly home (accompanied by an outpatient rehabilitation program). The latter patients usually leave the day after the chest tube has been removed, but others might wait much longer due to, among others, health insurance reasons. Therefore, we consider days with chest tube (or PAL) a better real-life indicator of postoperative course than length of hospital stay. There was evidence for an association between length of stay in hospital and the presence of adhesions (estimate, 3.83; 95% CI, 1.4-6.25; *P* = .002). However, the distribution of outcomes was not Gaussian and therefore the results need to be interpreted with caution.

We always considered terminating surgery in cases of severe adhesions to prevent prolonged length of hospital stay with no benefit, although we are not always confident that this is advantageous for the patient. However, based on the results of the present study, we will continue with unilateral surgery regardless of the type of PLA encountered. We usually perform bilateral surgery in patients with bilateral disease distribution; however, we now consider terminating the operation after the first side is completed if either severe PLA on the first side led to intraoperative air leak, or if severe PLA are discovered when exploring the second side. Notably, this practice arises from our experience and common sense and cannot be derived from the results of this study. Here, we show only that there is no correlation between PLA and postoperative improvement in FEV1%.

Because lung function improvement after unilateral LVRS is as effective as after bilateral LVRS, the latter patients might profit from the avoidance of PAL in terms of length of hospitalization. There is some debate in the literature on unilateral versus bilateral emphysema surgery, generally voting for a bilateral approach.^11^^,^^12^ Kotloff and colleagues^11^ compared 119 bilateral with 32 unilateral LVRS. Functional follow-up in 86 and 23 patients, respectively, showed a significant, but rather small, difference in FEV1 of 90 mL between the 2 groups, favoring the bilateral approach. The difference in 6-WMD was 195 feet (bilateral LVRS) compared with 147 feet (unilateral LVRS). However, of the 32 unilateral LVRS patients, 24 were part of a planned staged bilateral approach but only 10 received their contralateral operation; 10 were satisfied enough after the first side, 3 patients had a poor outcome with subsequent listing for transplantation, and 1 patient had intercurrent abdominal surgery.^11^

Argenziano and colleagues^12^ report significantly better improvements in spirometrics (FEV1 difference of 70% vs 28%) in bilateral surgery, but equal improvements in 6-WMD and dyspnea score in both procedures. This might reflect the difficulties of comparing often very heterogeneous groups undergoing unilateral versus bilateral operation in an nonprospective, randomized setting. A tendency toward better results after bilateral LVRS is usually reported in patients with upper lobe-predominant heterogeneous emphysema—a group in which we also usually favor the bilateral approach. In contrast, Oey and colleagues^13^^,^^14^ always vote for an unilateral approach. Implying enough benefit after 1 side, the second side might be spared treatment until lung function declines further. Nevertheless, many patients might get lost in-between, as COPD worsens and/or other comorbidities take their toll.

The results of this study do not show relevant outcome differences between uni- and bilateral operations, although almost all the unilateral operations were indicated unilaterally due to contraindications on the other side. Either there was unilateral disease, borderline inclusion criteria, a history of pleurodesis, or concomitant nodule/cancer resection. Only 12 patients actually had bilateral emphysema and were planned as bilateral LVRS, but were finally underwent operation on only 1 side due to adhesions or a massive air leak. Although this study was not intended to solve this issue, good results were confirmed in both unilateral and bilateral surgery. The interesting subgroup in which surgery was terminated prematurely cannot be assessed due to low numbers.

We chose COPD exacerbations as an outcome parameter because we saw no cases of postoperative pneumonia or empyema. Theoretically, PLA and prolonged length of hospital stay might predispose the vulnerable emphysema lung to exacerbations. This study shows a relatively high rate of postoperative COPD exacerbations (27.5%), but there was no evidence for a correlation of these PE with PLA.

The NETT trial reported a postoperative pneumonia rate of 18%.^1^ So far, there is no standardized approach for perioperative antibiotic prophylaxis in LVRS patients. We prefer broad-spectrum antibiotics as long as the chest tube is in situ—this might be questioned but might also explain our 0 rate of pneumonia or empyema in these patients.^15^

This study has some limitations. Its retrospective nature makes it prone to selection bias. Nevertheless, it was a consecutive observational cohort of all our LVRS patients over 4 years. PLA cannot be assessed definitively before surgery and therefore cannot be used as selection criteria (so far). A large limitation is the missing detailed information on the course of patients with PE. This potential complication after LVRS with PLA was more a guess than a known postoperative sequela but, lacking other infections, we consider it meaningful to check our patients for PE.

We have information only on spirometry, bodyplethysmography, and 6-MWD, and some postoperative values are missing. However, FEV1 seems to correlate well with both general condition and improvement.^16^, ^17^, ^18^ Nevertheless, more precise data, including questionnaires about quality of life and patient-reported outcome measures would be useful.^19^

Last, but not least, there is no standardized definition about the severity of PLA. Their influence on air leak—also in LVRS—has been demonstrated in the NETT data and now in this study as well.^7^ Regarding the surgical population in the NETT study, De Camp and colleagues^7^ reported none or minimal, moderate, or marked adhesions, with the latter 2 being found in 23% and 18% of cases, respectively. This might reflect our 24.6% rate of severe adhesions. Our definition of severe adhesion is as follows: severe dorsal adhesions potentially located also at the base of the upper lobe and at the lower lobe, therefore complicating easy resection or fistula closure after classical LVRS in an upper-lobe predominant emphysema (Figure 2). Apical adhesions (eg, potential parenchyma lesions) would be included in the LVRS specimen. The same might be true for severe mediastinal adhesions because these can be found at the lingula or anterior part of the right upper lobe; neither should be resected in the majority of LVRS procedures.

For our own practice, we consider complete adhesiolysis in patients scheduled for unilateral surgery because LVRS still has potential for lung function improvement despite PLA. In scheduled bilateral cases, we consider terminating the operation after successful LVRS on the first side if PLAs are detected on the second side.

## Conclusions

PLA might not influence the LFO of LVRS, although they can prolong chest tube time and, therefore, hospitalization time.

### Webcast

You can watch a Webcast of this AATS meeting presentation by going to: https://www.aats.org/resources/the-relevance-of-pleural-adhesions-for-the-short-and-long-term-outcome-of-lung-volume-reduction-surgery.

**Figure:**
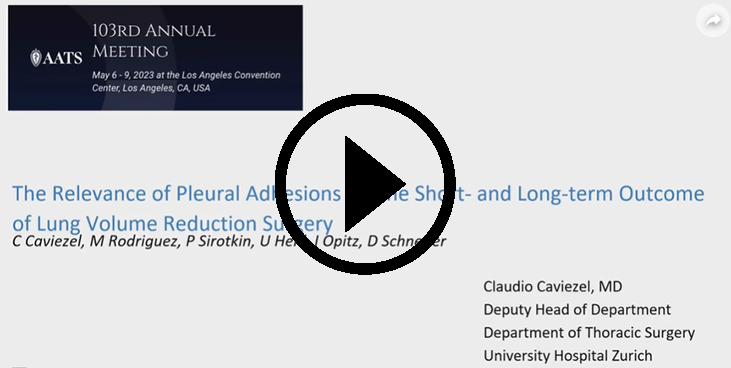

### Conflict of Interest Statement

The authors reported no conflicts of interest.

The *Journal* policy requires editors and reviewers to disclose conflicts of interest and to decline handling or reviewing manuscripts for which they may have a conflict of interest. The editors and reviewers of this article have no conflicts of interest.

## Supplementary Data

Video 1 Case report of unilateral LVRS in a patient with severe pleural adhesions. Video available at: https://www.jtcvs.org/article/S2666-2736(23)00179-1/fulltext.
