# Supplementary figures and images for: Relevance of pleural adhesions for short- and long-term outcomes after lung volume reduction surgery
Source: JTCVS Open. 2023 Jul 14;16:996–1003. doi: 10.1016/j.xjon.2023.06.018 (PMC10774947; doi:10.1016/j.xjon.2023.06.018)

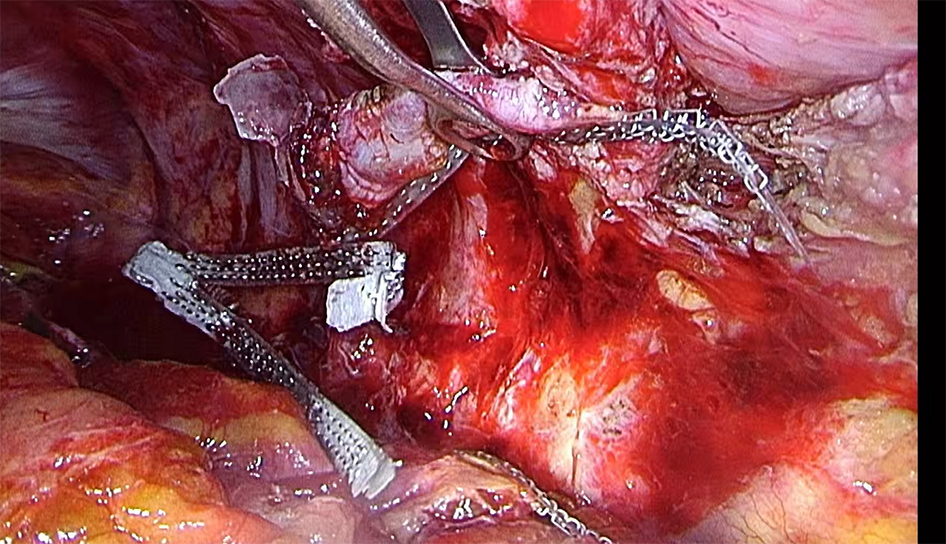

Supplement: Video 1 — Case report of unilateral LVRS in a patient with severe pleural adhesions. Video available at: https://www.jtcvs.org/article/S2666-2736(23)00179-1/fulltext. [file fx3.jpg]
